# News and Events of the Week

**Published:** 1910-07-09

**Authors:** 


### News and Events of the Week.

We have received from Messrs. John Walker and Co., Limited, of Warwick Lane, E.C., a copy of their Medical Loose-leaf Pocket-book. Planned to give the greatest amount of space possible without undue bulk, the book contains twelve different kinds of sheets, including those for temperatures, treatment, etc. An obstetrical table, a chart of treatment in cases of poisoning and sheets for prescriptions with the apparatus necessary for duplicating these are also provided. Individual requirements can be met by arrangement of the leaves, and if records are to be kept these can be filed in a transfer case without the trouble of re-writing. The price of the book is 8s. 6d. net.

British Association.—The acceptance of the invitation for the next meeting of the British Association for the Advancement of Science to be held in Australia would afford the greatest satisfaction locally (writes a Melbourne correspondent). Sir John Madden, Chancellor of the University of Melbourne, is chairman of the General Committee, that has given the invitation, and the Executive to attend to details has for its president Professor Masson, and as secretary Professor Spencer. The late Prime Minister, Mr. Deakin, promised pecuniary support. The State Governor, the Lord Mayor, members of Parliament, and the whole body of people approve of the proceeding.

A Meeting to discuss the aims and principles of the National Association for Providing Rational Curative Treatment for Alcoholism and the Drug Habit was held at 18 Collingham Place, S.W., the residence of the Bishop of Croydon, on June 27. The association, which is purely philanthropic, is under most influential patronage. A number of representative people were present. The association aims at establishing a Home Hospital in London for educated women workers suffering from alcoholism and the drug habit, and £1,100 is needed for this. The association's address is 65 Upper Gloucester Place, N.W. All inquiries will be gladly replied to on application to the Hon. Secretary.

Sydney Holds her "Hospital Sunday" in May.—On the first day of the month 5,000 collectors came forward to help, among them Miss Lily Brayton, Mr. Oscar Asche, and the members of their company. Miss Brayton sold a number of "buttonholes" for 5s. The amount was the largest ever collected; from street collections alone the sum was £6,808. The Government of Victoria has asked Dr. Burnett Ham, chairman of the Board of Public Health, to undertake investigations as to the prevalence of specific (venereal) diseases in the community. These have been made notifiable within the metropolitan area. Medical men are to inform the Board of any cases that come under their observation, with the age and sex only of the sufferer; a specimen of the blood to be sent to the University. For these offices the doctors are paid. Later on may or should follow the establishment of a special hospital with its own laboratory.

At the annual outing of Coleman & Co., Ltd., the proprietors of Wincarnis, the firm's representative outlined a scheme of pension and invalidity insurance which it has been decided to adopt on behalf of the employees. Employees above sixty-five years who have served the company for at least ten years will receive a pension not exceeding 5s. a week. Those of fifteen years' standing will get 6s., and those of twenty years' standing, 7s. 6d. a week. Any person eligible for a pension and wishing to resign his position in the company must apply to the committee for the grant of a pension. A pension may be given for the life of the pensioner or for such period as the committee, with the approval of the trustees, may determine, but a person whose pension has expired may be re-elected.

Syme Prize for Research.—The David Syme Prize, given by the proprietors of the *Age* newspaper, consists of a gold medal and £100 cash. It is awarded for the best thesis based upon original work in biology, chemistry, geology or natural philosophy, and, other things being equal, preference is given to research connected with the material and industrial development of Australia. It is open to all science workers in Australia, except professors and heads of Government departments. That for 1910 has just been awarded to Dr. H. G. Chapman for his researches on the precipitin reaction. Dr. Chapman, a native-born Australian, is a graduate of the University of Melbourne, and is at present Demonstrator of Physiology at the University of Sydney. Besides the present subject, Dr. Chapman has conducted researches on neurology and pharmacology.

The Metropolitan Asylums Board is amongst the least advertised of the great public bodies of London. The immense work that it carries out is not confined to dealing with infectious diseases, but includes also asylum treatment of imbeciles, the treatment of Poor Law children, especially for ringworm, ophthalmia, and debility; the maintenance of homes and one training ship for juvenile offenders; and an ambulance service comprising eight stations and some scores of ambulance vehicles and three riverside wharves. From a monetary point of view the Board is in a satisfactory position, having done an increased amount of work during the year 1909 with a diminished expenditure of £44,164 and with a simultaneous reduction of its indebtedness by a sum of £184,399. The Asylums bulletin for 1909, the twelfth year since the present form of issue was begun, consists of reports from a large number of different committees and statistical tables that will be of interest chiefly to those concerned with public health. In addition to this, there is a medical supplement, consisting of forty pages, dealing with subjects which are of direct interest to all practitioners, and including accounts of complications of the various infectious diseases; post-scarlatinal diphtheria; tracheotomy and intubation in laryngeal diphtheria; the results of laparotomy in cases of typhoid fever with perforation; and original articles upon Vincent's angina.

The new Prayer-book, with the change of names necessitated by the Accession of King George, was ready actually last week. Messrs Eyre & Spottiswoode are to be congratulated on the promptitude with which its publication followed the Order in Council, which was received by them only a few hours before.

Dr. Edward Charles Bury, of York Row, Wisbech, medical officer of health for the Marshland and Wisbech Rural Districts, surgeon to the North Cambridgeshire Hospital and the Wisbech Fever Hospital, who died on April 23, aged seventy-five, left estate of the value of £22,104 gross and £2,885 net.

The Council of the Royal College of Physicians of Edinburgh has awarded to Dr. R. W. Philip, F.R.C.P., physician to the Edinburgh Royal Infirmary and to the Royal Victoria Consumption Hospital, the Cullen Victoria Jubilee Prize for his investigations and work in connection with consumption. The award is made every four years for meritorious work in medical science, and is to the value of £100.

The newly established experimental dental clinic opened its doors to patients last Thursday at 213 Shaftesbury Avenue, Holborn, W.C. The only qualification for treatment is ability to pay self-supporting fees and inability to pay the ordinary fees of dentists. It will be remembered that £200,000 was offered in April last to supply London first and then provincial cities with dental aid services for the class with the above qualification.

Visit of American Doctors to London.—The American doctors who have come to this country for the purpose of studying hospital surgical methods have now visited most of the London institutions. They watched Sir W. Watson Cheyne's operations at King's College, and were much interested in the antiseptic methods employed by this distinguished surgeon. On Tuesday last they visited the House of Commons, where they were received by Sir William Collins and the other medical members of the House, and took tea on the terrace after listening to the debates.

The Council of the Society for the Study of Inebriety has decided that in view of the annual meeting in London during July of the British Medical Association, it will be inexpedient to hold any formal summer meeting. The July meeting will, therefore, not take place this year. On Tuesday, October 11, 1910 (afternoon meeting), John Newton, Esq., Secretary of the United Committee for the Prevention of the Demoralisation of Native Races by the Liquor Traffic, will open a discussion on "Alcohol and Primitive Races." The meeting will be held in the rooms of the Medical Society, 11 Chandos Street, W.

A well-known Paris doctor, the executor of the will of the late François Coppée, M. Duchastelet, was killed on July 4 in Paris, says the *Times* correspondent, in a singular motor-car accident. He had passed the evening at one of the Champs Elysées music-halls. On coming away at 11.30 he was standing in front of his electric motor-car, lighting the lantern, when suddenly the car started forward of its own motion, and crushed Dr. Duchastelet against another car that was just behind him. He fainted and was carried to the Beaujon Hospital, where he died shortly afterwards. The only possible explanation which has been thus far vouchsafed of this accident is that a drop of rain fell on the rheostat of the dynamo while the doctor was standing in front of the motor-car and that it produced a short circuit, which set into simultaneous action the entire series of electric batteries, with the result that the car started off at its highest speed.

